# Supplementary material for: Incidence and predictive biomarkers of Clostridioides difficile infection in hospitalized patients receiving broad-spectrum antibiotics
Source: Nat Commun. 2021 Apr 14;12:2240. doi: 10.1038/s41467-021-22269-y (PMC8046770; doi:10.1038/s41467-021-22269-y)
Supplement: Supplementary file 1 — Supplementary Information [file 41467_2021_22269_MOESM1_ESM.pdf]

Supplementary Information

Incidence and predictive biomarkers of *Clostridioides difficile* infection in hospitalized patients receiving broad-spectrum antibiotics

Table of Contents

Supplementary methods: Ethics committees 3
Supplementary methods: Sample collection and processing 4
Figure S1: Sample collection scheme 4
Toxigenic *C. difficile* rectal carriage 4
16S rRNA gene profiling 5
Normalized urinary 3-indoxyl sulfate 5
CDI diagnostic testing 5
Supplementary Methods: Multiple imputation 6
Original planned imputation method 6
Problems identified during imputation 6
AAD event occurring during initial hospitalization 6
Timing of AAD episode 6
Figure S2: Stool test missingness by onset day of AAD event 7
Figure S3: Stool test positivity by onset day of AAD event 8
Country 8
Table S1: Stool sample testing and positivity by country 8
Possible overfitting problem of the imputation model due to data sparsity 9
Supplementary results 10
Table S2: Number of participants and observed CDI episodes by country and site 10
Normalized 3-indoxyl sulfate 11
Table S3: Median [IQR] N3-IS for subjects developing CDI, AAD without CDI, and no AAD 11
Supplementary results for CDI 12
Table S4: Time to first observed CDI 12
Table S5: AIC, performance and external validation of OTU ratio models for CDI 13

Table S6: AIC, performance and external validation of OTU abundance models for CDI 14
Table S7: Univariable stratified incidence of CDI within 28 days - sensitivity analysis with unimputed data 15
Table S8: Univariable stratified incidence of CDI within 28 days - sensitivity analysis with complete cases 16
Table S9: Univariable stratified incidence of CDI within 28 days - sensitivity analysis restricted to countries with at least 75% of AAD episodes tested for CDI 17
Table S10: Univariable stratified incidence of CDI within 90 days - sensitivity analysis with unimputed data 18
Table S11: Univariable stratified incidence of CDI within 90 days - sensitivity analysis with complete cases 19
Table S12: Univariable stratified incidence of CDI within 90 days - sensitivity analysis restricted to countries with at least 75% of AAD episodes tested for CDI 20
Supplementary results for AAD 21
Table S13: AIC of top-10 OTU-ratio and top-20 OTU-abundance models for AAD within 90 days 21
Table S14: Univariable stratified incidence of AAD within 28 days - sensitivity analysis with complete cases 22
Table S15: Univariable stratified incidence of AAD within 90 days - sensitivity analysis with complete cases 23
References 24

Supplementary methods: Ethics committees

Netherlands: local medical ethics committee approval

- UMC Utrecht

Germany: local medical ethics committee approvals

- UKK Uniklinik Köln
- Universitätsklinikum Heidelberg (KLIPPS)
- Jena University Hospital
- UK-SH (UZL) Universitätsklinikum Schleswig-Holstein, Campus Lübeck
- Klinikum der Universität München
- Universitätsklinikum Leibzig
- University of Aachen
- Universitatsklinikum Essen

Greece: local medical ethics committee approvals

- University Hospital of Heraklion
- Laiko General Hospital
- Attikon University General Hospital
- Evangelismos General Hospital of Athens
- Ippokrateio General Hospital of Athens

Spain: Central approvals:

- Comité Coordinador de Ética de la Investigación Biomédica de Andalucía
- Dirección General de Inspección y Ordenación CONSEJERÍA DE SANIDAD Comunidad de Madrid

Local medical ethics committee approvals

- Hospital Universitari de Bellvitge
- Hospital Universitario 12 de Octubre
- Hospital Universitario Gregorio Marañon
- Hospital Universitario Ramon y Cajal
- Hospital Universitario Virgen Macarena
- Hospital Universitari Vall d'Hebrón
- Servicio Andaluz de Salud- Reina Sofia University Hospital

Romania: Central approval:

- Ministry of Health, National Agency for Medicines and Medical Devices

Local medical ethics committee approvals

- Infectious and Tropical Diseases Hospital “Dr. Victor Babes”
- Clinical Hospital Of Infectious Diseases Of Iasi
- The National Institute of Infectious Diseases Matei Bals

- Cluj Napoca Infectious disease Clinical Hospital
- Oncology Institute Ion Chiricuta

France: Central approvals:

- ANSM (Agance nationale de sécurité du médicament et des produits de santé)
- Comite de protection des personnes du Sud-Ouest et outre-mer IV, Limoges

Supplementary methods: Sample collection and processing

During the enrolment visit, between 72 hours before and 6 hours after initiation of antibiotic treatment, a urine sample for normalized 3-indoxyl sulfate (N3-IS) measurement, a rectal swab for 16S rRNA gene profiling, and a rectal swab for *C. difficile* carriage detection by PCR were collected. At D6 +/- 1 (or D3 or D4 if the participant's discharge was planned prior to D5), another urine sample for N3-IS measurement and another rectal swab for 16S rRNA profiling were collected. Urine was collected either by spontaneous urine production by the participant or by taking urine through a bladder catheter being in place for medical reasons. Participants were not catheterized for obtaining urine for research purposes only. FecalSwab^{TM} Regular Flocked Collection Kit with universal transport medium was used to collect material for 16S rRNA gene profiling. A flocked swab in a dry tube was used to collect material for identification of *C. difficile* carriage by PCR which was previously validated for this purpose (1). Samples were frozen at -80 degrees C within 4 hours of collection and were shipped on frozen carbon dioxide (dry ice) in batches to the central laboratory for analysis.

Supplementary Figure 1: Sample collection scheme

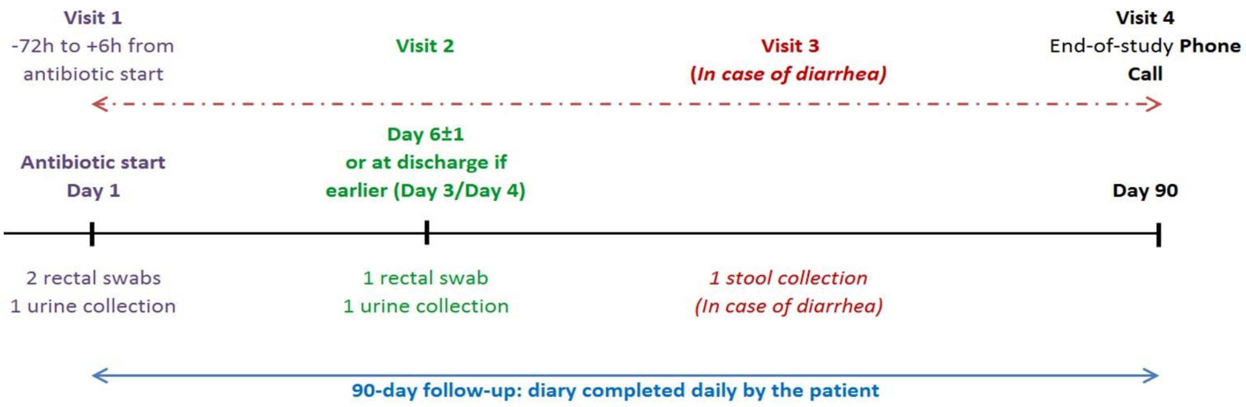

Toxigenic *C. difficile* rectal carriage

Flocked swabs were tested by the GeneXpert *C. difficile* test (Cepheid), a multiplex real-time PCR that detects the toxin B gene (tcdB), the binary toxin gene (cdt) and the TcdC gene deletion at nt117 indicating the hypervirulent *C. difficile* ribotype 027. The test was performed according to the manufacturer's instructions except that rectal swabs were used instead of stool samples. The swabs

were discharged in the sample reagent. The results were reported as positive, negative, or invalid for each target. As a quality control, a randomly selected 10% of the results data were cross-checked by a second person.

16S rRNA gene profiling

Rectal swabs were sent to the local laboratories within 2 hours after collection and stored at -80°C until shipment to the Central Laboratory in dry ice conditions. A detailed description of 16S rRNA gene profiling is provided elsewhere (2).

Normalized urinary 3-indoxyl sulfate

Urine collectors were sent to the local laboratories within 2 hours after collection to be aliquoted. All prepared aliquots were stored without delay at -80°C until shipment to the Central Laboratory in dry ice conditions. To quantify 3-IS levels, 200 µL IS working standard solution was pipetted in 20 µL urine. After short vortex mixing the sample was centrifuged for 10 minutes at 20,500 x*g* (14,000 RPM). The supernatant was transferred to a clean vial and 2-10 µL was injected into a High Performance Liquid Chromatography Tandem Mass Spectrometry system. An API-4000 triple quadrupole tandem mass spectrometer (Sciex/ Applied Biosystems) was used as the detector. The range of quantification of 3-IS in human urine is approximately 4.00 to 40000 nmol/L. Urine samples were analysed for Creatinine concentrations using an enzymatic method on the COBAS8000. N3-IS levels were expressed as nmol 3-IS/ µmol creatinine and categorized as low (log2(N3-IS) < -8), intermediate (-8 <= log2(N3-IS) <- 2.5), or high (log2(N3-IS) > 2.5) as used previously (3).

CDI diagnostic testing

In addition to collection of scheduled samples, an additional fresh faecal sample was collected, if participants reported diarrhea during the 90-day follow-up period. In each country, one hospital was appointed as central study laboratory for diagnosis of CDI. Faecal samples were kept under refrigerator conditions and shipped to the central laboratory within 72 hours of collection. Samples were tested according to one of the two testing algorithms proposed by the ESCMID guideline on diagnosis of CDI (4). Results were reported back to the hospital that enrolled the participant. Management decisions related to the CDI test result were left to the discretion of the treating physician.

Supplementary Methods: Multiple imputation

Multiple imputation was performed using the *mice* package version 3.3.0.

Original planned imputation method

Missing data were expected for the outcome of AAD or CDI (for instance, due to withdrawal, loss to follow-up, or the diarrhea sample is missing). To compensate for differences in follow-up duration, time to event analysis (Cox regression) was applied with censoring at the end of follow-up. Missing CDI diagnoses in case of diarrhea due to not testing a stool sample was imputed. This imputation was planned to be performed at the level of the diarrhea episode, taking into account the baseline characteristics, number of previous diarrhea and CDI episodes, and time to the diarrhea episode. If needed and achievable, the type of diagnostic test that should have been performed (PCR or GDH and type of confirmation test) was planned to be included in the imputation algorithm. We also planned to test the assumption of non-informative censoring.

Problems identified during imputation

Upon imputation it was observed that for CDI very high positivity rates were imputed. For those with AAD with missing stool test results, the imputed CDI rate was approx. twice as high as for those with AAD with CDI test results available. This was partly explained by patterns in the data and partly by what is possibly an overfitting problem of the imputation model due to data sparsity, as explained below.

AAD event occurring during initial hospitalization

Of 80 AAD events occurring during initial hospitalization, CDI results were unknown in 13 (16%). Of 67 with valid testing performed, 7 (10%) were positive.

Of 92 AAD events occurring after the initial hospitalization, CDI results were unknown in 42 (46%). Of 50 with valid testing performed, 10 (20%) were positive.

(4 AAD events had unknown timing with respect to discharge date or episode date, of which CDI results were available in 1 [0 CDI positive].)

This was suggestive of missing not at random. Therefore, it was decided to not include in the imputation model whether the event occurred during the initial admission.

Timing of AAD episode

Stool samples were more often missed late in follow-up (Figure 3.0.0.1), but if tested, they were slightly often positive (Figure 3.0.0.2). This is probably related to whether the patient was hospitalized, hence, this variable was also not included in the imputation model.

Supplementary Figure 2: Stool test missingness by onset day of AAD event

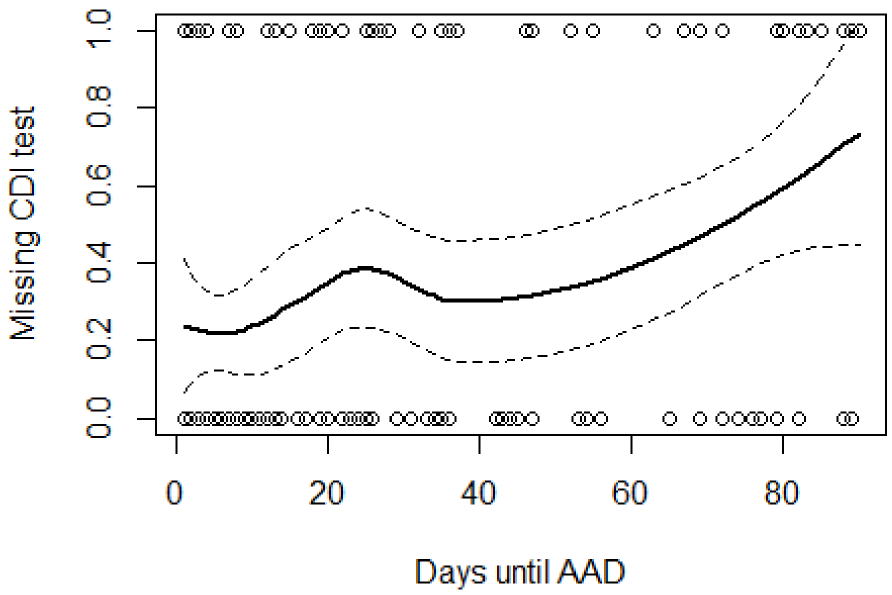

Abbreviations: AAD: antibiotic-associated diarrhea, CDI: *Clostridioides difficile* infection.

Supplementary Figure 3: Stool test positivity by onset day of AAD event

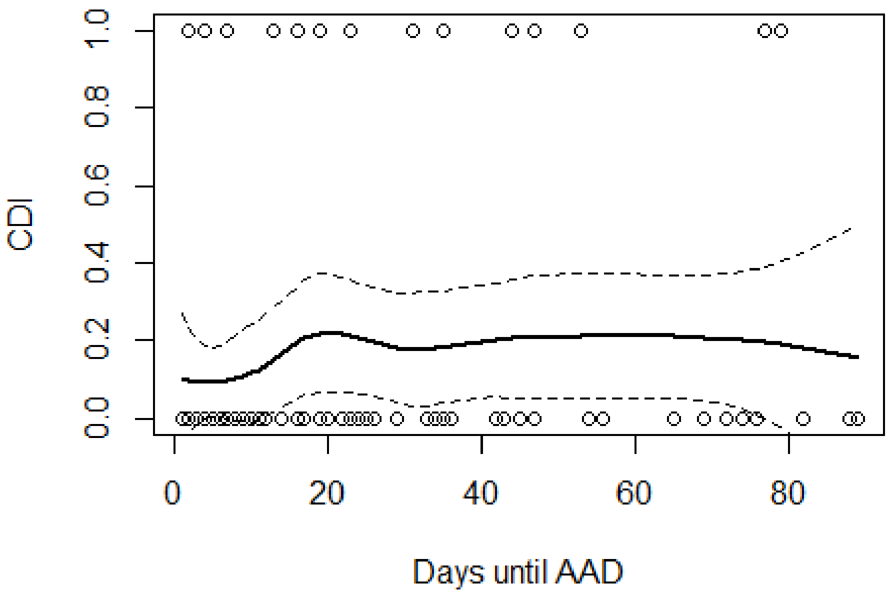

Abbreviations: AAD: antibiotic-associated diarrhea, CDI: *Clostridioides difficile* infection.

Country

Romania contributed 43% of missing samples but if tested, samples were positive in 45% of episodes (Table 3.0.0.1). This was also suggestive of missing not at random. Therefore, it was decided to also not include in the imputation model whether the event had occurred during the initial admission.

Supplementary Table 1: Stool sample testing and positivity by country

Country	AAD episodes	Valid test performed	CDI positive *
France	25	14 (56%)	0 (0%)
Germany	50	46 (92%)	8 (17%)
Greece	24	18 (75%)	1 (6%)
Netherlands	8	4 (50%)	0 (0%)
Romania	36	11 (31%)	5 (45%)
Spain	33	25 (76%)	3 (12%)

* % of episodes in which valid testing was performed.

Abbreviations: AAD: antibiotic-associated diarrhea, CDI: *Clostridioides difficile* infection.

The testing algorithm was, as per the study design, related to the country (GR and RO used the GHD-based testing algorithm). Therefore, the original plan to include the diagnostic testing algorithm (PCR or GDH) in the imputation algorithm was also dismissed.

Possible overfitting problem of the imputation model due to data sparsity

The data patterns did not fully explain the higher than expected imputed rates of CDI. It was, therefore, suspected that the imputation model was overfitted due to data sparsity. This was confirmed by running data simulations with one variable with the level of missing values comparable to CDI status (i.e. 948 without CDI, 15 with CDI, and 44 missing) and an increasing number of randomly generated variables. These simulations showed that, under these conditions, as the number of predictors increased, the imputed proportion in those with a missing value also increased. The number of required predictors for imputing CDI was minimized to those that were considered biologically important. Subsequently it was determined that the inflation factor was 2.4. Therefore, in a second imputation the probability of imputing CDI was reduced by a factor 0.42. In simulations, the second (i.e. corrected) imputation model turned out to more reliably provide the imputed incidence while correlations were maintained. Therefore, this approach was used in the final analysis. Also, sensitivity analyses were performed after excluding the countries with low testing rate (NL, RO, and FR). Data were separately imputed for this analysis as missing data patterns might be different. In this dataset, there were only 10 CDI episodes and 16 missing, and the estimated correction needed was slightly higher (reduction by factor 0.37).

Supplementary results

Supplementary Table 2: Number of participants and observed CDI episodes by country and site

Country / site	Number of enrolled participants	Number of observed CDI episodes
**Netherlands:**	**15**	**0**
Site 10	15	0
**Germany:**	**159**	**7**
Site 20	83	6
Site 21	3	0
Site 22	21	0
Site 23	18	0
Site 24	21	1
Site 25	5	0
Site 26	3	0
Site 27	5	0
**Greece**	**85**	**1**
Site 30	24	0
Site 31	22	0
Site 32	32	0
Site 33	4	1
Site 34	3	0
**Spain**	**331**	**2**
Site 40	30	0
Site 41	10	0
Site 42	17	0
Site 43	6	1
Site 44	98	1
Site 45	11	0
Site 46	159	0
**Romania**	**189**	**5**
Site 50	16	1
Site 51	8	0
Site 52	35	1
Site 53	37	0
Site 54	93	3
**France**	**228**	**0**
Site 60	19	0
Site 61	20	0
Site 62	38	0
Site 63	21	0

Site 64	26	0
Site 65	19	0
Site 66	80	0
Site 67	5	0

Normalized 3-indoxyl sulfate

Supplementary Table 3: Median [IQR] N3-IS for subjects developing CDI, AAD without CDI, and no AAD

Subgroup	Baseline N3-IS	Day-6 N3-IS*	Difference in N3-IS†
N	938	784	762
No AAD	26.8 [16.9 – 39.8]	18.1 [7.0 – 31.6]	-8.1 [-19.2 – 2.0]
AAD (including CDI)	25.5 [17.8 – 39.0]	9.8 [0.6 – 23.3]	-13.2 [-25.5 – -2.5]
No CDI	26.7 [16.9 – 39.9]	17.2 [5.7 – 31.0]	-8.5 [-19.8 – 1.6]
CDI	30.5 [23.6 – 38.2]	3.9 [1.4 – 10.3]	-21.1 [-31.1 – -14.8]

* In subjects with no CDI prior to the Day-6 measurement. † In subjects with baseline N3-IS measurement and no CDI prior to the D6 measurement.

Abbreviations: AAD: antibiotic associated diarrhea, CDI: *C. difficile* infection, IQR: inter-quartile range, N3-IS: normalized 3-indoxyl sulfate.

Supplementary results for CDI

Supplementary Table 4: Time to first observed CDI

Population	Events	Median (IQR)	% within 28 days	% within 45 days
Total population	15	18 (4-38)	60%	80%
Penicillin + BLI	5	15 (3-18)	80%	80%
3rd or 4th generation cephalosporin	6	24 (9-33)	50%	100%
Fluoroquinolone	2	32 (22-42)	50%	50%
Carbapenem	4	23 (10-45)	50%	75%
Clindamycin	0	-	-	-
History of CDI	0	-	-	-
No history of CDI	14	18 (4-41)	57%	79%
Toxigenic *C. difficile* carriage at baseline	4	2 (1-3)	100%	100%
No toxigenic *C. difficile* carriage at baseline	11	30 (16-44)	45%	73%
High N3-IS level	15	18 (4-38)	60%	80%
Intermediate N3-IS level	0	-	-	-
Low Shannon index	10	16 (5-42)	60%	70%
High Shannon index	4	20 (5-36)	50%	100%
Low inverse Simpson index	10	16 (5-42)	60%	70%
High inverse Simpson index	4	20 (5-36)	50%	100%

Abbreviations: BLI: beta-lactamase inhibitor, CDI: *Clostridioides difficile* infection, IQR: inter-quartile range, N3-IS: normalized 3-indoxyl sulfate.

Supplementary Table 5: AIC, performance and external validation of OTU ratio models for CDI

High risk definition	Derivation study (ANTICIPATE)					Validation study (Vincent 2013)			
	**AIC**	**Prevalence**	**Incidence low**	**Incidence high**	**SDHR**	**Prevalence**	**Incidence low**	**Incidence high**	**RR**
OTU1 / OTU648 ≥ 26	226.1	20.6%	1.0% (0.3-2.4)	5.4% (1.5-13.4)	**5.8 (2.1-20.8)**	21.1%	3.7% (1.8-7.1)	9.9% (3.8-33.1)	2.7 (0.8-10.6)
OTU1 / OTU31 ≥ 8.5	227.8	16.9%	1.1% (0.4-2.4)	5.7% (1.8-13.5)	**5.4 (2.1-18.7)**	13.5%	3.4% (1.7-6.2)	15.5% (5.2-100.0)	**4.6 (1.4-28.8)**
OTU1 / OTU280 ≥ 4.3	230.1	38.3%	0.7% (0.1-2.4)	3.8% (0.7-13.2)	**6.2 (1.6-34.2)**	49.4%	2.5% (0.7-5.8)	7.6% (3.9-15.8)	**3.1 (1.0-13.3)**
OTU1 / OTU30 ≥ 1.6	230.2	24.5%	1.0% (0.3-2.4)	4.6% (1.1-12.7)	**4.9 (1.8-17.0)**	53.6%	1.8% (0.3-5.2)	7.8% (4.1-15.5)	**4.3 (1.3-29.2)**
OTU1 / OTU69 ≥ 26	231.2	25.5%	1.0% (0.4-2.3)	4.5% (1.0-12.6)	**4.7 (1.8-16.2)**	*			
OTU1 / OTU68 ≥ 1.7	234.0	35.8%	0.9% (0.3-2.5)	3.7% (0.6-12.7)	**4.4 (1.4-16.7)**	37.5%	2.7% (1.0-5.7)	8.9% (4.2-20.4)	**3.3 (1.1-12.6)**
OTU1 / OTU56 ≥ 5.8	234.2	31.1%	1.0% (0.3-2.4)	3.8% (0.7-12.4)	**4.1 (1.5-13.7)**	*			

Numbers between brackets denote 95% confidence intervals. Statistically significant SDHR and RR are indicated in bold. Prevalence: proportion of participants meeting the risk group definition. Only models that were significant in de derivation dataset are provided.

* These models could not be validated because OTU56 and OTU69 were not identified in the validation set.

Abbreviations: AIC: Akaikes Information Criterion, CDI: *Clostridioides difficile* infection, OTU: operational taxonomic unit, RR: relative risk, SDHR: sub-distribution hazard ratio, OTU1: *Enterococcus*. OTU30: *Blautia*. OTU31: *Ruminococcus.* OTU56: *Porphyromonas*. OTU68: *Alistipes*. OTU69: *Porphyromonas.* OTU280: *Bifidobacterium*. OTU648: *Blautia.*

Supplementary Table 6: AIC, performance and external validation of OTU abundance models for CDI

High risk definition	Derivation study (ANTICIPATE)					Validation study (Vincent 2013)			
	AIC	Prevalence	Incidence low	Incidence high	SDHR	Prevalence	Incidence low	Incidence high	RR
OTU1 ≥ 0.087% & OTU30 < 0.093%	219.2	12.9%	1.0% (0.4-2.3)	7.8% (3.6-14.9)	**7.5 (2.6-30.3)**	44.7%	3.8% (1.5-8.2)	6.5% (3.0-14.1)	1.7 (0.6-5.8)
OTU30 < 0.093% & OTU56 < 0.293%	222.6	14.0%	1.0% (0.4-2.3)	7.3% (3.2-14.2)	**7.0 (2.4-26.8)**	*			
OTU30 < 0.093% & OTU69 < 0.013%	224.4	12.1%	1.1% (0.5-2.3)	7.6% (3.4-14.7)	**6.6 (2.5-21.9)**	*			
OTU1 ≥ 0.087% & OTU648 < 0.047%	226.2	20.8%	0.9% (0.3-2.2)	5.6% (2.3-11.6)	**6.3 (2.2-24.0)**	41.3%	2.8% (1.0-6.3)	8.1% (3.8-18.0)	2.8 (0.9-10.4)
OTU1 ≥ 0.087% & OTU68 < 0.087%	227.5	17.9%	1.1% (0.4-2.3)	5.8% (2.3-11.8)	**5.4 (2.0-19.5)**	13.9%	2.9% (1.3-5.5)	18.0% (6.8-100)	**6.2 (2.0-34.8)**
OTU1 ≥ 0.087% & OTU9 < 1.320%	228.6	20.3%	1.1% (0.4-2.4)	5.0% (1.9-11.0)	**4.5 (1.8-14.8)**	18.3%	2.3% (0.9-4.6)	17.1% (7.3-51.1)	**7.4 (2.5-35.9)**
OTU1 ≥ 0.087% & OTU280 < 0.073%	228.8	28.3%	0.9% (0.3-2.3)	4.4% (1.5-10.3)	**4.8 (1.8-17.3)**	46.0%	1.5% (0.3-4.1)	9.1% (4.6-18.6)	**5.9 (1.8-37.4)**
OTU1 ≥ 0.087% & OTU69 < 0.013%	229.3	22.8%	1.0% (0.4-2.3)	4.9% (1.8-10.9)	**4.8 (1.8-16.2)**	*			
OTU1 ≥ 0.087% & OTU31 < 0.043%	229.5	13.2%	1.3% (0.6-2.5)	6.0% (2.5-12.3)	**4.7 (1.9-15.3)**	6.1%	2.9% (1.4-5.5)	37.7% (12.1-100)	**13.0 (3.7-59.7)**
OTU1 ≥ 0.087% & OTU56 < 0.293%	229.6	29.8%	0.9% (0.3-2.3)	4.2% (1.4-10.2)	**4.8 (1.7-18.2)**	*			

Numbers between brackets denote 95% confidence intervals. Statistically significant SDHR and RR are indicated in bold. Prevalence: proportion of participants meeting the risk group definition. Only the first 10 models, ranked by AIC, are provided.

* These models could not be validated because OTU56 and OTU69 were not identified in the validation set.

Abbreviations: AIC: Akaikes Information Criterion, CDI: *Clostridioides difficile* infection, OTU: operational taxonomic unit, RR: relative risk, SDHR: sub-distribution hazard ratio, OTU1: *Enterococcus*. OTU9: *Finegoldia*. OTU30: *Blautia*. OTU31: *Ruminococcus.* OTU56: *Porphyromonas*. OTU68: *Alistipes*. OTU69: *Porphyromonas.* OTU280: *Bifidobacterium*. OTU648: *Blautia.*

Supplementary Table 7: Univariable stratified incidence of CDI within 28 days - sensitivity analysis with unimputed data

Subgroup	N	Events	Incidence	SDHR
Penicillin + BLI	409	4	1.0% (0.3-2.5)	1.2 (0.3-4.4)
3rd /4th gen cephalosporin	394	3	0.8% (0.2-2.2)	0.8 (0.2-3.1)
Fluoroquinolone	181	1	0.6% (0.1-3.0)	0.6 (0.1-4.5)
Carbapenem	64	2	3.2% (0.6-10.0)	4.2 (0.9-20.1)
Clindamycin	29	0	-	-
History of CDI	14	0	-	-
No history of CDI	907	8	0.9% (0.4-1.7)	-
History of CDI unknown	86	1	1.2% (0.1-6.0)	-
Toxigenic *C. difficile* carriage (GeneXpert, Cepheid)	35	4	11.4% (3.5-24.5)	**22.5 (6.0-84.3)**
No toxigenic *C. difficile* carriage	948	5	0.6% (0.2-1.3)	-
Toxigenic *C. difficile* carriage unknown	24	0	-	-
Intermediate N3-IS level ($-8 \leq \log2(\text{N3-IS}) \leq 2.5$)	28	0	-	-
High N3-IS level ($\log2(\text{N3-IS}) > 2.5$)	910	9	1.0% (0.5-1.9)	-
N3-IS level unknown	69	0	-	-
Shannon index ≤ 2.586	131	6	4.7% (1.9-9.5)	**18.7 (3.8-92.8)**
Shannon index > 2.586	814	2	0.3% (0.1-0.9)	-
Inverse Simpson index ≤ 7.674	185	6	3.4% (1.4-6.9)	**12.5 (2.5-62.4)**
Inverse Simpson index > 7.674	760	2	0.3% (0.1-0.9)	-

Missing or invalid CDI test results are considered as negative. Subjects starting combination treatment are included in multiple antibiotic subgroups. Incidence derived from the Cumulative Incidence Function with death as competing event. For antibiotic classes the SDHR is relative to patients not receiving that antibiotic class. Abbreviations: BLI: beta-lactamase inhibitor, CDI: *Clostridioides difficile* infection, N3-IS: Normalized 3-indoxyl sulfate levels, SDHR: subdistribution hazard ratio.

Supplementary Table 8: Univariable stratified incidence of CDI within 28 days - sensitivity analysis with complete cases

Subgroup	N	Events	Incidence	SDHR
Penicillin + BLI	399	4	1.1% (0.3-2.5)	1.2 (0.3-4.4)
3rd /4th gen cephalosporin	387	3	0.8% (0.2-2.2)	0.8 (0.2-3.0)
Fluoroquinolone	172	1	0.6% (0.1-3.2)	0.6 (0.1-4.7)
Carbapenem	62	2	3.3% (0.6-10.3)	4.2 (0.9-20.2)
Clindamycin	28	0	-	-
History of CDI	13	0	-	-
No history of CDI	887	8	0.9% (0.4-1.8)	-
History of CDI unknown	80	1	1.3% (0.1-6.5)	-
Toxigenic *C. difficile* carriage (GeneXpert, Cepheid)	33	4	12.1% (3.7-25.8)	**23.4 (6.2-87.4)**
No toxigenic *C. difficile* carriage	924	5	0.6% (0.2-1.3)	-
Toxigenic *C. difficile* carriage unknown	23	0	-	-
Intermediate N3-IS level ($-8 \leq \log2(\text{N3-IS}) \leq 2.5$)	27	0	-	-
High N3-IS level ($\log2(\text{N3-IS}) > 2.5$)	885	9	1.1% (0.5-2.0)	-
N3-IS level unknown	68	0	-	-
Shannon index $\leq$ 2.586	126	6	4.9% (2.0-9.8)	**18.5 (3.7-91.8)**
Shannon index > 2.586	775	2	0.3% (0.1-0.9)	-
Inverse Simpson index $\leq$ 7.674	179	6	3.5% (1.5-7.1)	**12.3 (2.5-61.2)**
Inverse Simpson index > 7.674	722	2	0.3% (0.1-1.0)	-

Patients with missing or invalid CDI test results are excluded from this analysis. Subjects starting combination treatment are included in multiple antibiotic subgroups. Incidence derived from the Cummulative Incidence Function with death as competing event. For antibiotic classes the SDHR is relative to patients not receiving that antibiotic class.

Abbreviations: BLI: beta-lactamase inhibitor, CDI: *Clostridioides difficile* infection, N3-IS: Normalized 3-indoxyl sulfate levels, SDHR: subdistribution hazard ratio.

Supplementary Table 9: Univariable stratified incidence of CDI within 28 days - sensitivity analysis restricted to countries with at least 75% of AAD episodes tested for CDI

Subgroup	N	Events	Incidence	SDHR
Penicillin + BLI	298	4.1	1.4% (0.5-3.4)	3.4 (0.4-30.4)
3rd /4th gen cephalosporin	152	0.1	-	-
Fluoroquinolone	119	0.0	-	-
Carbapenem	42	1.0	2.4% (0.2-10.9)	3.0 (0.3-26.5)
Clindamycin	5	0.0	-	-
History of CDI	20	0.0	-	-
No history of CDI	555	5.2	1.0% (0.4-2.2)	-
Toxigenic *C. difficile* carriage (GeneXpert, Cepheid)	28	3.0	11.0% (2.6-26.2)	**28.5 (4.8-170.8)**
No toxigenic *C. difficile* carriage	547	2.2	0.4% (0.1-1.5)	-
Intermediate N3-IS level ($-8 \leq \log2(\text{N3-IS}) \leq 2.5$)	21	0.0	-	-
High N3-IS level ($\log2(\text{N3-IS}) > 2.5$)	554	5.2	1.0% (0.4-2.2)	-
Shannon index $\leq$ 2.586	99	4.6	4.8% (1.6-10.7)	-
Shannon index > 2.586	476	0.6	0.1% (0.0-1.7)	-
Inverse Simpson index $\leq$ 7.674	133	4.6	3.5% (1.2-7.9)	-
Inverse Simpson index > 7.674	442	0.6	0.1% (0.00-1.9)	-

Patients from countries with <75% of diarrhea episodes correctly tested for CDI test were excluded from this analysis. Included are Germany, Greece and Spain. Subjects starting combination treatment are included in multiple antibiotic subgroups. Incidence derived from the Cummulative Incidence Function with death as competing event. For antibiotic classes the SDHR is relative to patients not receiving that antibiotic class.

Abbreviations: BLI: beta-lactamase inhibitor, CDI: *Clostridioides difficile* infection, N3-IS: Normalized 3-indoxyl sulfate levels, SDHR: subdistribution hazard ratio.

Supplementary Table 10: Univariable stratified incidence of CDI within 90 days - sensitivity analysis with unimputed data

Subgroup	N	Events	Incidence	SDHR
Penicillin + BLI	409	5	1.3% (0.5-2.9)	0.7 (0.2-2.2)
3rd /4th gen cephalosporin	394	6	1.6% (0.7-3.3)	1.0 (0.4-2.9)
Fluoroquinolone	181	2	1.2% (0.2-3.9)	0.7 (0.2-3.1)
Carbapenem	64	4	6.5% (2.1-14.5)	**5.3 (1.7-16.6)**
Clindamycin	29	0	-	-
History of CDI	14	0	-	-
No history of CDI	907	14	1.6% (0.9-2.7)	-
History of CDI unknown	86	1	1.2% (0.1-6.0)	-
Toxigenic *C. difficile* carriage (GeneXpert, Cepheid)	35	4	11.4% (3.5-24.5)	**10.3 (3.2-33.1)**
No toxigenic *C. difficile* carriage	948	11	1.3% (0.7-2.2)	-
Toxigenic *C. difficile* carriage unknown	24	0	-	-
Intermediate N3-IS level (-8 ≤ log2(N3-IS) ≤ 2.5)	28	0	-	-
High N3-IS level (log2(N3-IS) > 2.5)	910	15	1.7% (1.0-2.8)	-
N3-IS level unknown	69	0	-	-
Shannon index ≤ 2.586	131	10	8.0% (4.1-13.7)	**16.0 (5.0-50.8)**
Shannon index > 2.586	814	4	0.5% (0.2-1.3)	-
Inverse Simpson index ≤ 7.674	185	10	5.8% (3.0-10.0)	**10.7 (3.3-34.0)**
Inverse Simpson index > 7.674	760	4	0.6% (0.2-1.4)	
Ratio OTU1/OTU31 ≥ 8.5 *	147	10	5.6% (1.8-13.1)	**7.0 (2.7-24.7)**
Ratio OTU1/OTU31 < 8.5	798	4	0.8% (0.3-1.8)	
OTU1 >= 0.087% & OTU68 < 0.087% *	163	10	5.2% (1.2-14.8)	**5.9 (1.9-20.4)**
OTU1 < 0.087% OR OTU68 ≥ 0.087%	782	4	0.8% (0.3-1.9)	

Missing or invalid CDI test results are considered as negative. Subjects starting combination treatment are included in multiple antibiotic subgroups. Incidence derived from the Cummulative Incidence Function with death as competing event. For antibiotic classes the SDHR is relative to patients not receiving that antibiotic class. Abbreviations: BLI: beta-lactamase inhibitor, CDI: *Clostridioides difficile* infection, N3-IS: Normalized 3-indoxyl sulfate levels, OTU1: *Enterococcus*, OTU31: *Ruminococcus*, OTU68: *Alistipes*, SDHR: subdistribution hazard ratio.
* Bias-adjusted incidences and SDHR are provided.

Supplementary Table 11: Univariable stratified incidence of CDI within 90 days - sensitivity analysis with complete cases

Subgroup	N	Events	Incidence	SDHR
Penicillin + BLI	397	5	1.3% (0.5-2.9)	0.7 (0.2-2.1)
3rd /4th gen cephalosporin	375	6	1.7% (0.7-3.5)	1.0 (0.4-2.9)
Fluoroquinolone	169	2	1.3% (0.2-4.2)	0.7 (0.2-3.2)
Carbapenem	59	4	7.1% (2.2-15.7)	**5.5 (1.8-17.3)**
Clindamycin	28	0	-	-
History of CDI	12	0	-	-
No history of CDI	872	14	1.7% (1.0-2.8)	-
History of CDI unknown	79	1	1.4% (0.1-6.5)	-
Toxigenic *C. difficile* carriage (GeneXpert, Cepheid)	32	4	12.5% (3.9-26.5)	**10.8 (3.4-35.0)**
No toxigenic *C. difficile* carriage	908	11	1.3% (0.7-2.3)	-
Toxigenic *C. difficile* carriage unknown	23	0	-	-
Intermediate N3-IS level (-8 ≤ log2(N3-IS) ≤ 2.5)	27	0	-	-
High N3-IS level (log2(N3-IS) > 2.5)	870	15	1.8% (1.1-2.9)	-
N3-IS level unknown	66	0	-	-
Shannon index ≤ 2.586	126	10	8.4% (4.3-14.2)	**15.8 (4.9-50.2)**
Shannon index > 2.586	775	4	0.6% (0.2-1.3)	-
Inverse Simpson index ≤ 7.674	179	10	6.0% (3.1-10.4)	**10.5 (3.3-33.4)**
Inverse Simpson index > 7.674	722	4	0.6% (0.2-1.4)	-
Ratio OTU1/OTU31 ≥ 8.5 *	139	10	5.9% (2.3-12.3)	**7.1 (2.6-25.5)**
Ratio OTU1/OTU31 < 8.5	762	4	0.8% (0.3-2.0)	
OTU1 >= 0.087% & OTU68 < 0.087% *	153	10	5.5% (1.4-15.5)	**6.0 (1.9-22.4)**
OTU1 < 0.087% OR OTU68 ≥ 0.087%	748	4	0.8% (0.3-2.0)	

Patients with missing or invalid CDI test results are excluded from this analysis. Subjects starting combination treatment are included in multiple antibiotic subgroups. Incidence derived from the Cummulative Incidence Function with death as competing event. For antibiotic classes the SDHR is relative to patients not receiving that antibiotic class.

Abbreviations: BLI: beta-lactamase inhibitor, CDI: *Clostridioides difficile* infection, N3-IS: Normalized 3-indoxyl sulfate levels, OTU1: *Enterococcus*, OTU31: *Ruminococcus*, OTU68: *Alistipes*, SDHR: subdistribution hazard ratio.

* Bias-adjusted incidences and SDHR are provided.

Supplementary Table 12: Univariable stratified incidence of CDI within 90 days - sensitivity analysis restricted to countries with at least 75% of AAD episodes tested for CDI

Subgroup	N	Events	Incidence	SDHR
Penicillin + BLI	298	5.1	1.8% (0.7-3.9)	0.9 (0.3-3.1)
3rd /4th gen cephalosporin	152	2.3	1.6% (0.3-5.2)	0.8 (0.2-3.6)
Fluoroquinolone	119	1.0	0.9% (0.1-4.7)	0.4 (0.1-3.3)
Carbapenem	42	2.2	5.3% (0.9-15.7)	3.2 (0.7-14.9)
Clindamycin	5	0.0	-	-
History of CDI	20	0.2	-	-
No history of CDI	555	10.2	1.9% (1.0-3.4)	-
Toxigenic *C. difficile* carriage (GeneXpert, Cepheid)	28	3.2	11.7% (2.8-27.7)	**9.2 (2.3-36.5)**
No toxigenic *C. difficile* carriage	547	7.2	1.4% (0.6-2.8)	-
Intermediate N3-IS level ($-8 \leq \log2(\text{N3-IS}) \leq 2.5$)	21	0.0	-	-
High N3-IS level ($\log2(\text{N3-IS}) > 2.5$)	554	10.4	2.0% (1.0-3.5)	-
Shannon index $\leq$ 2.586	99	8.8	9.3% (4.2-16.9)	**28.3 (4.2-188.4)**
Shannon index > 2.586	476	1.7	0.4% (0.1-1.6)	-
Inverse Simpson index $\leq$ 7.674	133	8.8	6.9% (3.2-12.5)	**19.9 (2.8-138.6)**
Inverse Simpson index > 7.674	442	1.6	0.4% (0.1-1.7)	-
Ratio OTU1/OTU31 $\geq$ 8.5 *	105	7.5	5.9% (0.7-22.1)	**6.0 (1.9-29.5)**
Ratio OTU1/OTU31 < 8.5	470	3.0	1.0% (0.3-2.8)	
OTU1 >= 0.087% & OTU68 < 0.087% *	104	7.4	5.9% (0.3-29.8)	**4.4 (1.4-21.1)**
OTU1 < 0.087% OR OTU68 $\geq$ 0.087%	471	3.0	1.0% (0.3-3.1)	

Patients from countries with <75% of diarrhea episodes correctly tested for CDI test were excluded from this analysis. Included are Germany, Greece and Spain. Subjects starting combination treatment are included in multiple antibiotic subgroups. Incidence derived from the Cumulative Incidence Function with death as competing event. For antibiotic classes the SDHR is relative to patients not receiving that antibiotic class.

Abbreviations: BLI: beta-lactamase inhibitor. CDI: *Clostridioides difficile* infection. N3-IS: Normalized 3-indoxyl sulfate levels, OTU1: *Enterococcus*, OTU31: *Ruminococcus*, OTU68: *Alistipes*, SDHR: subdistribution hazard ratio.

* Bias-adjusted incidences and SDHR are provided.

Supplementary results for AAD

Supplementary Table 13: AIC of top-10 OTU-ratio and top-20 OTU-abundance models for AAD within 90 days

Model	AIC
OTU-ratio models	
1. OTU21 / OTU648 *	1819.33
2. OTU1 / OTU69	1819.39
3. OTU1 / OTU127	1820.87
4. OTU21 / OTU2540	1821.39
5. OTU1392 / OTU69	1821.69
6. OTU1 / OTU92	1822.75
7. OTU1392 / OTU127	1822.85
8. OTU1 / OTU64	1823.52
9. OTU1392 / OTU648	1823.73
10. OTU1392 / OTU56	1824.18
OTU-abundance models	
1. OTU69 & OTU21 & OTU648 & OTU37 & OTU18	1805.68
2. OTU69 & OTU21 & OTU648 & OTU37 & OTU92	1806.36
3. OTU69 & OTU21 & OTU648 & OTU37 & OTU1	1806.47
4. OTU69 & OTU21 & OTU648 & OTU37 & OTU25	1806.88
5. OTU69 & OTU21 & OTU648 & OTU37 & OTU127	1806.91
6. OTU69 & OTU21 & OTU648 & OTU37 & OTU2540	1807.04
7. OTU69 & OTU21 & OTU648 & OTU37 & OTU1392	1807.54
8. OTU69 & OTU21 & OTU648 & OTU37 *	1807.61
9. OTU69 & OTU21 & OTU648 & OTU37 & OTU56	1807.89
10. OTU69 & OTU21 & OTU648 & OTU92	1808.53
11. OTU69 & OTU21 & OTU648 & OTU18	1809.12
12. OTU69 & OTU21 & OTU648 & OTU37 & OTU64	1809.36
13. OTU69 & OTU21 & OTU648 & OTU1	1809.59
14. OTU69 & OTU21 & OTU648 & OTU25	1810.01
15. OTU69 & OTU21 & OTU648 & OTU56	1810.03
16. OTU69 & OTU21 & OTU648 & OTU127	1810.48
17. OTU69 & OTU21 & OTU648 & OTU2540	1810.74
18. OTU69 & OTU21 & OTU648 & OTU1392	1810.92
19. OTU69 & OTU21 & OTU648	1811.50
20. OTU69 & OTU21 & OTU648 & OTU64	1812.65

* Selected as the best model. For the OTU-abundance model, OTU37 improved the overall model fit, but the optimal breakpoint was indifferent of the OTU37 value. Therefore, OTU37 was ignored in the final model. Abbreviations: AAD: antibiotic associated diarrhea, AIC: Akaikes Information Criterion,.OTU1: *Enterococcus*, OTU18: *Prevotella*, OTU21: uncultured *Lachnospiraceae*, OTU25: *Campylobacter*, OTU37: uncultured *Clostridiales*, OTU56: *Porphyromonas*, OTU64: *Ezakiella*, OTU69: *Porphyromonas*, OTU92: *Dialister*, OTU127: *Oscillibacter*, OTU648: *Blautia*, OTU1392: *Clostridium* cluster XlVa, OTU2540: *Ruminococcus*.

Supplementary Table 14: Univariable stratified incidence of AAD within 28 days - sensitivity analysis with complete cases

Subgroup	N	Events	Incidence	SDHR
Penicillin + BLI	409	45	11.5% (8.5-14.8)	1.2 (0.8-1.8)
3rd /4th gen cephalosporin	394	30	7.6% (5.2-10.6)	**0.6 (0.4-1.0)**
Fluoroquinolone	181	18	10.4% (6.4-15.5)	1.0 (0.6-1.6)
Carbapenem	64	15	24.1% (14.3-35.3)	**2.8 (1.6-4.8)**
Clindamycin	29	2	7.6% (1.3-21.8)	-
History of CDI	14	2	15.4% (2.2-39.8)	1.7 (0.4-6.8)
No history of CDI	907	88	10.0% (8.1-12.1)	-
History of CDI unknown	86	11	13.1% (6.9-21.3)	-
Toxigenic *C. difficile* carriage (GeneXpert, Cepheid)	35	7	20.0% (8.7-34.7)	2.2 (1.0-4.7)
No toxigenic *C. difficile* carriage	948	92	10.0% (8.1-12.0)	-
Toxigenic *C. difficile* carriage unknown	24	2	9.8% (1.6-27.3)	-
Intermediate N3-IS level ($-8 \leq \log2(\text{N3-IS}) \leq 2.5$)	28	2	7.1% (1.2-20.7)	0.7 (0.2-2.8)
High N3-IS level ($\log2(\text{N3-IS}) > 2.5$)	910	94	10.6% (8.7-12.8)	-
N3-IS level unknown	69	5	7.8% (2.8-16.1)	-
Shannon index $\leq$ 3.155	363	45	12.8% (9.6-16.6)	1.5 (1.0-2.2)
Shannon index > 3.155	579	49	8.8% (6.6-11.4)	-
Inverse Simpson index $\leq$ 14.339	485	54	11.5% (8.8-14.6)	1.3 (0.8-1.9)
Inverse Simpson index > 14.339	457	40	9.1% (6.7-12.1)	-

Abbreviations: BLI: beta-lactamase inhibitor, CDI: *Clostridioides difficile* infection, N3-IS: normalized 3-indoxyl sulfate.

Supplementary Table 15: Univariable stratified incidence of AAD within 90 days - sensitivity analysis with complete cases

Subgroup	N	Events	Incidence	SDHR
Penicillin + BLI	409	51	13.1% (9.9-16.6)	0.9 (0.6-1.3)
3rd /4th gen cephalosporin	394	47	12.6% (9.4-16.2)	0.8 (0.6-1.1)
Fluoroquinolone	181	25	14.6% (9.8-20.4)	1.0 (0. 7-1.6)
Carbapenem	64	21	33.9% (22.3-45.7)	**3.0 (1.9-4.7)**
Clindamycin	29	3	11.4% (2.8-26.8)	-
History of CDI	14	2	15.4% (2.2-39.8)	1.2 (0.3-5.1)
No history of CDI	907	121	14.1% (11.8-16.5)	-
History of CDI unknown	86	12	14.4% (7.8-22.8)	-
Toxigenic *C. difficile* carriage (GeneXpert, Cepheid)	35	7	20.0% (8.7-34.7)	1.6 (0.7-3.5)
No toxigenic *C. difficile* carriage	948	125	13.9% (11.7-16.2)	-
Toxigenic *C. difficile* carriage unknown	24	3	15.1% (3.5-34.3)	-
Intermediate N3-IS level (-8 ≤ log2(N3-IS) ≤ 2.5)	28	3	10.7% (2.6-25.4)	0.7 (0.2-2.3)
High N3-IS level (log2(N3-IS) > 2.5)	910	125	14.5% (12.2-16.9)	-
N3-IS level unknown	69	7	11.4% (4.9-20.8)	-
Shannon index ≤ 3.155	363	62	17.9% (14.1-22.2)	**1.6 (1.1-2.2)**
Shannon index > 3.155	579	64	11.6% (9.1-14.5)	-
Inverse Simpson index ≤ 14.339	485	74	16.0% (12.8-19.5)	1.4 (0.9-1.9)
Inverse Simpson index > 14.339	457	52	12.0% (9.1-15.3)	-
Ratio OTU21/OTU648 ≥ 6.3 *	206	44	20.2% (15.1-26.0)	**1.7 (1.3-2.4)**
Ratio OTU21/OTU648 < 6.3	736	82	12.3% (9.8-15.2)	
At least two of OTU69 < 0.140% or OTU21 ≥ 0.013% or OTU648 < 0.006% *	376	74	18.2% (14.3-22.8)	**1.7 (1.3-2.2)**
Less than two of OTU69 < 0.140% or OTU21 ≥ 0.013% or OTU648 < 0.006%	566	52	11.2% (8.7-14.0)	

Abbreviations: AAD: Antibiotic associated diarrhea, BLI: beta-lactamase inhibitor, CDI: *Clostridioides difficile* infection, N3-IS: normalized 3-indoxyl sulfate, OTU21: uncultured *Lachnospiraceae*, OTU69: *Porphyromonas*, OTU648: *Blautia*, SDHR: subdistribution hazard ratio.

* Bias-adjusted incidences and SDHR are provided.

References

1. Jazmati, N., Kirpal, E., Piepenbrock, E., Stelzer, Y., Vehreschild, M.J.G.T. & Seifert, H. Evaluation of the Use of Rectal Swabs for Laboratory Diagnosis of Clostridium difficile Infection. *J Clin Microbiol* **56**, e00426-18 (2018).

2. Berkell, M., et al. Microbiota-based markers predictive of development of Clostridioides difficile infection. *Nature communications* (2021).

3. Weber, D., et al. Low urinary indoxyl sulfate levels early after transplantation reflect a disrupted microbiome and are associated with poor outcome. *Blood* **126**, 1723-1728 (2015).

4. Crobach, M.J.T., et al. European Society of Clinical Microbiology and Infectious Diseases: update of the diagnostic guidance document for Clostridium difficile infection. *Clin Microbiol Infect* **22**, Suppl 4 S63–81 (2016).
