# Supplementary material for: Incidence and predictive biomarkers of Clostridioides difficile infection in hospitalized patients receiving broad-spectrum antibiotics
Source: Nat Commun. 2021 Apr 14;12:2240. doi: 10.1038/s41467-021-22269-y (PMC8046770; doi:10.1038/s41467-021-22269-y)
Supplement: Supplementary file 3 — Reporting Summary [file 41467_2021_22269_MOESM3_ESM.pdf]

nature research

Corresponding author(s): Cornelis H. van Werkhoven
Maria J.G.T. Vehreschild

Last updated by author(s): Jun 18, 2020

Reporting Summary

Nature Research wishes to improve the reproducibility of the work that we publish. This form provides structure for consistency and transparency in reporting. For further information on Nature Research policies, see our Editorial Policies and the Editorial Policy Checklist.

Statistics

For all statistical analyses, confirm that the following items are present in the figure legend, table legend, main text, or Methods section.

n/a	Confirmed	
☐	☒	The exact sample size (*n*) for each experimental group/condition, given as a discrete number and unit of measurement
☐	☒	A statement on whether measurements were taken from distinct samples or whether the same sample was measured repeatedly
☐	☒	The statistical test(s) used AND whether they are one- or two-sided<br>*Only common tests should be described solely by name; describe more complex techniques in the Methods section.*
☐	☒	A description of all covariates tested
☐	☒	A description of any assumptions or corrections, such as tests of normality and adjustment for multiple comparisons
☐	☒	A full description of the statistical parameters including central tendency (e.g. means) or other basic estimates (e.g. regression coefficient) AND variation (e.g. standard deviation) or associated estimates of uncertainty (e.g. confidence intervals)
☐	☒	For null hypothesis testing, the test statistic (e.g. *F*, *t*, *r*) with confidence intervals, effect sizes, degrees of freedom and *P* value noted<br>*Give P values as exact values whenever suitable.*
☒	☐	For Bayesian analysis, information on the choice of priors and Markov chain Monte Carlo settings
☒	☐	For hierarchical and complex designs, identification of the appropriate level for tests and full reporting of outcomes
☐	☒	Estimates of effect sizes (e.g. Cohen's *d*, Pearson's *r*), indicating how they were calculated

Our web collection on statistics for biologists contains articles on many of the points above.

Software and code

Policy information about availability of computer code

Data collection	Clinical data was entered electronically into Research Online 2, a Remote Data Capture tool developed by the University Medical Center Utrecht, see https://www.researchonline.info/en-us/ABOUT-US for more information. Clostridioides difficile infection was determined according to the algorithm provided in the ESCMID diagnostic guidance document for Clostridium difficile infection (July 22, 2016, DOI:https://doi.org/10.1016/j.cmi.2016.03.010).
Data analysis	Data were analysed in R version 3.5.1 (2018-07-02). The following R packages were used: mice version 3.3.0 (multiple imputation), OptimalCutpoints version 1.1-3 (Youden index to determine optimal cutoff), cmprsk version 2.2-7 (Fine & Gray competing risks model), pROC version 1.13.0 (calculation of C-statistic).

For manuscripts utilizing custom algorithms or software that are central to the research but not yet described in published literature, software must be made available to editors and reviewers. We strongly encourage code deposition in a community repository (e.g. GitHub). See the Nature Research guidelines for submitting code & software for further information.

Data

Policy information about availability of data

All manuscripts must include a data availability statement. This statement should provide the following information, where applicable:

- Accession codes, unique identifiers, or web links for publicly available datasets
- A list of figures that have associated raw data
- A description of any restrictions on data availability

Sequence data collected in this study has been made available at the Sequence Read Archive under project accession number PRJNA685914. Access to clinical data is restricted as the informed consent provided does not allow for use of clinical data outside the research institution.

Field-specific reporting

Please select the one below that is the best fit for your research. If you are not sure, read the appropriate sections before making your selection.

☒ Life sciences ☐ Behavioural & social sciences ☐ Ecological, evolutionary & environmental sciences

For a reference copy of the document with all sections, see nature.com/documents/nr-reporting-summary-flat.pdf

Life sciences study design

All studies must disclose on these points even when the disclosure is negative.

Sample size	The sample size was chosen such that the impact of randomly over- or underestimating the incidence would be acceptably low. Assuming an event rate of approximately 2% in the total included population, 839 participants would be needed to accurately determine the event rate. Assuming an event rate of 5% in one of the subgroups, 335 participants would be needed in that subgroup to accurately determine the event rate in the high-risk group. If one third of participants were part of such a high-risk population, this would require a total study population of 1,005 patients. Therefore, we aimed to include 1,000 participants into the study. This sample size was deemed sufficient to perform a robust sample size calculation for an RCT.
Data exclusions	In- and exclusion criteria were prespecified. Of patients meeting the eligiblity criteria, none were excluded.
Replication	Not applicable, not an experimental study.
Randomization	Not applicable, not an experimental study.
Blinding	Blinded assessment was not deemed necessary given objective outcome definitions.

Reporting for specific materials, systems and methods

We require information from authors about some types of materials, experimental systems and methods used in many studies. Here, indicate whether each material, system or method listed is relevant to your study. If you are not sure if a list item applies to your research, read the appropriate section before selecting a response.

Materials & experimental systems

n/a	Involved in the study	
☒	☐	Antibodies
☒	☐	Eukaryotic cell lines
☒	☐	Palaeontology and archaeology
☒	☐	Animals and other organisms
☐	☒	Human research participants
☐	☒	Clinical data
☒	☐	Dual use research of concern

Methods

n/a	Involved in the study	
☒	☐	ChIP-seq
☒	☐	Flow cytometry
☒	☐	MRI-based neuroimaging

Human research participants

Policy information about studies involving human research participants

Population characteristics	Median age was 70 years (IQR 62-79), 592 participants (58.8%) were male. The Charlson comorbidity index was median 5.1 [IQR 3.8 - 6.8]. Prevalence of comorbidities: Cardiovascular disease: 31.9%, Hematological/oncological disease: 33.8%, Diabetes mellitus: 27.9%, COPD: 15.9%, Gastrointestinal disease: 15.5%, Moderate to severe chronic kidney disease: 13.1, history of CDI: 1.5. Concomitant medication in past 3 months: Systemic antibiotics: 42.0%, immunosuppressants: 23.2, proton pump inhibitors: 50.9.
Recruitment	Pharmacy registries or admission lists of hospitalized patients were screened for eligibility during office hours. Patients meeting the inclusion criteria were approached for written informed consent, which was obtained prior to any study related procedures. Selection bias may have occurred as a result of inability or refusal to provide informed consent. As a result, the disease severity of the population may have been underestimated, potentially leading to a lower incidence of CDI in our population.
Ethics oversight	The study protocol was approved by a central ethics review board in each country and/or the local institutional review boards of each hospital, in accordance with the local regulations.<br>Netherlands: local medical ethics committee approval<br>• UMC Utrecht<br>Germany: local medical ethics committee approvals<br>• UKK Uniklinik Köln<br>• Universitätsklinikum Heidelberg (KLIPPS)<br>• Jena University Hospital<br>• UK-SH (UZL) Universitätsklinikum Schleswig-Holstein, Campus Lübeck<br>• Klinikum der Universität München<br>• Universitätsklinikum Leibzig<br>• University of Aachen<br>• Universitatsklinikum Essen<br>Greece: local medical ethics committee approvals<br>• University Hospital of Heraklion<br>• Laiko General Hospital<br>• Attikon University General Hospital<br>• Evangelismos General Hospital of Athens<br>• Ippokrateio General Hospital of Athens<br>Spain: Central approvals:<br>• Comité Coordinador de Ética de la Investigación Biomédica de Andalucía<br>• Dirección General de Inspección y Ordenación CONSEJERÍA DE SANIDAD Comunidad de Madrid<br>Local medical ethics committee approvals<br>• Hospital Universitari de Bellvitge<br>• Hospital Universitario 12 de Octubre<br>• Hospital Universitario Gregorio Marañon<br>• Hospital Universitario Ramon y Cajal<br>• Hospital Universitario Virgen Macarena<br>• Hospital Universitari Vall d'Hebrón<br>• Servicio Andaluz de Salud- Reina Sofia University Hospital<br>Romania: Central approval:<br>• Ministry of Health, National Agency for Medicines and Medical Devices<br>Local medical ethics committee approvals<br>• Infectious and Tropical Diseases Hospital "Dr. Victor Babes"<br>• Clinical Hospital Of Infectious Diseases Of Iasi<br>• The National Institute of Infectious Diseases Matei Bals<br>• Cluj Napoca Infectious disease Clinical Hospital<br>• Oncology Institute Ion Chiricuta<br>France: Central approvals:<br>• ANSM (Agance nationale de sécurité du médicament et des produits de santé)<br>• Comite de protection des personnes du Sud-Ouest et outre-mer IV, Limoges

Note that full information on the approval of the study protocol must also be provided in the manuscript.

Clinical data

Policy information about clinical studies

All manuscripts should comply with the ICMJEguidelines for publication of clinical research and a completedCONSORT checklist must be included with all submissions.

Clinical trial registration	ClinicalTrials.gov: NCT02896244
Study protocol	The study protocol has been uploaded as supplemental file.
Data collection	This was a multicenter prospective observational cohort study in 34 hospitals (21 university and 13 non-university hospitals) from

France, Germany, Greece, the Netherlands, Romania and Spain. Patients were recruited from September 2016 through October 2017. Data was collected concerning a followup period per participant of 90 days.

Outcomes

The primary endpoint was CDI within 28 days after initiation of antibiotic treatment. An episode of CDI was defined as a clinical picture compatible with CDI and microbiological evidence of free toxins and the presence of C. difficile in stools, using one of the two algorithms recommended in the 2016 European Society of Clinical Microbiology and Infectious Diseases (ESCMID) guideline, without reasonable evidence 338 of another cause of diarrhea, or pseudomembranous colitis as diagnosed during endoscopy, after colectomy or on autopsy. Diarrhea was defined as loose stools, i.e. taking the shape of the receptacle, corresponding to Bristol stool chart types 5-7, and a stool frequency of at least three in 24 consecutive hours. Secondary endpoints included incidence of CDI within 90 days (assessed in the same way as the primary endpoint) and incidence of AAD within 28 and 90 days (defined as three or more loose stools [i.e. taking the shape of the receptable] within 24 hours as reported by the participant).
